# Phenotype–environment matching in sand fleas

**DOI:** 10.1098/rsbl.2015.0494

**Published:** 2015-08

**Authors:** Martin Stevens, Annette C. Broderick, Brendan J. Godley, Alice E. Lown, Jolyon Troscianko, Nicola Weber, Sam B. Weber

**Affiliations:** 1 Centre for Ecology & Conservation, College of Life & Environmental Sciences, University of Exeter, Penryn Campus, Penryn, Cornwall TR10 9FE, UK; 2 Conservation Office, Ascension Island Government, Georgetown, Ascension Island, ASCN 1ZZ, South Atlantic Ocean

**Keywords:** camouflage, phenotype–environment, predation, coloration

## Abstract

Camouflage is perhaps the most widespread anti-predator strategy in nature, found in numerous animal groups. A long-standing prediction is that individuals should have camouflage tuned to the visual backgrounds where they live. However, while several studies have demonstrated phenotype–environment associations, few have directly shown that this confers an improvement in camouflage, particularly with respect to predator vision. Here, we show that an intertidal crustacean, the sand flea (*Hippa testudinaria*), has coloration tuned to the different substrates on which it occurs when viewed by potential avian predators. Individual sand fleas from a small, oceanic island (Ascension) matched the colour and luminance of their own beaches more closely than neighbouring beaches to a model of avian vision. Based on past work, this phenotype–environment matching is likely to be driven through ontogenetic changes rather than genetic adaptation. Our work provides some of the first direct evidence that animal coloration is tuned to provide camouflage to prospective predators against a range of visual backgrounds, in a population of animals occurring over a small geographical range.

## 1. Introduction

Camouflage is ubiquitous in nature and a major strategy for avoiding being seen by both predators and prey. Much recent research has sought to understand the mechanistic basis of how camouflage types work and their relative value, in both real animals and artificial systems [1]. However, progress in testing camouflage matching of animals to different natural backgrounds has been slower. One major prediction is that there should be an association between the appearance of animals and the backgrounds where they live, something appreciated since Wallace [2]. Specifically, individuals within a given habitat should show phenotype–environment matches, whereby their appearance is tuned to provide concealment against their relevant visual background. Such changes could be driven by genetic adaptation over generations [3], phenotypic plasticity [4,5], or both.

To date, most research has demonstrated phenotype–environment associations but not matching [4–6]. That is, research has shown associations between aspects of animal appearance and different habitats, but not directly demonstrated that camouflage itself is enhanced against the relevant substrates where an animal lives, as opposed to alternative backgrounds. In addition, most studies have relied on human assessments of appearance, or have yet to consider predator vision. Some of the best candidates to use to explore these questions are marine arthropods, which live on a variety of visually distinct backgrounds. For example, previous work by Wenner [7] described evidence that individual Pacific mole crabs (Decapoda; ‘sand fleas’), *Hippa pacifica*, had appearances in line with the coloration of Hawaiian beaches. Here, we test whether individual sand fleas of *Hippa testudinaria*, found on different beaches of Ascension Island (with sand of substantially different appearance [8]), match their respective backgrounds to predator (avian) vision. Sand fleas occupy the swash zone of sandy beaches, emerging from the sediment to feed, during which time they are likely to be exposed to various visually guided predators, such as shorebirds and crabs [9].

## 2. Methods

Sand fleas were collected in February 2013 from 13 different beaches on Ascension Island corresponding to a range of visual appearances (see electronic supplementary material, figure S1). Collection was based on opportunistically capturing individuals within the narrow swash zone, where they are accessible. Exact sampling locations were chosen haphazardly depending on the distribution of sand fleas within each beach. Individuals were frozen and flown directly back to the UK (while stored on ice), and transported to laboratories at the University of Exeter until analysis. Any individuals that showed signs of decay were not analysed. There were no obvious indications of appearance change owing to the freezing process. Samples of sand from each location were also collected. Sand fleas sample sizes were 19–20 individuals for most beaches, but some locations had fewer (electronic supplementary material).

Camouflage assessment was based on digital image analysis, following a range of past methods [10,11]. Briefly, sand fleas were allowed to thaw and immediately photographed in a dark room under an ultraviolet and human visible Arc Lamp (Iwasaki EYE Color Arc Lamp with its UV filter removed) diffused with a silver photographic umbrella. To photograph the substrate, samples were saturated with water (to match where the fleas were collected) and spread out in a flat container. All images were taken from a standardized distance and angle with a Nikon D7000 digital camera, which had undergone a quartz conversion to enable ultraviolet (UV) sensitivity (Advanced Camera Services, UK), and fitted with a Nikon 105 mm Nikkor lens. A UV/IR blocking filter was used for the human visible photos, transmitting wavelengths of 400–700 nm (Baader UV/IR Cut Filter). A UV pass and IR blocking filter was used for the ultraviolet photographs (Baader U filter; transmitting between 300 and 400 nm). This resulted in five image layers: longwave (LW), mediumwave (MW), shortwave (SW) and two ultraviolet (UV) layers (from the red and blue channels). Each image included a Spectralon reflectance standard (Labsphere, Congleton, UK) reflecting light at 40% between 300 and 700 nm.

Following photography, using custom scripts in ImageJ [12], images were linearized and standardized to the standard to remove the effects of illuminating light [11]. Images were then transformed to correspond to an avian visual system using a mapping technique that is highly accurate compared with spectrometry-based vision modelling [10–12]. Birds have four single cone types used in colour vision (sensitive to LW, MW, SW and UV light), and additional double cones that seem to be used in luminance vision. Although Ascension has no resident breeding shorebirds (vagrants occur), they are a likely predator group providing the original selection pressure for camouflage in sand fleas [9]. Shore birds have reduced UV sensitivity [13], and we therefore used the visual sensitivity of the peafowl (*Pavo cristatus*; [14]), a model species for this type of bird vision, to generate images with predicted avian cone catch values for the LW, MW, SW, UV and double cone types.

Following image processing, each individual was selected in ImageJ (avoiding areas of specular reflectance where light ‘bounces’ back from the sample surface), and the five cone catch values were measured. In addition, image samples of a standardized area of sand from each beach were measured (haphazardly selected from the substrate samples; *n* = number of corresponding sand fleas from the same beach). We then used a widely implemented model of visual discrimination [15] to compare the colour and luminance (lightness) of each sand flea with one random sample of their own background, and with a random sample from each of the other beaches. We used the log form of the tetrachromatic version of the Vorobyev–Osorio model, which assumes that receptor noise limits visual discrimination, with a Weber fraction value of 0.05 for the most abundant cone type, and relative proportions of cone types of the peafowl (LW = 0.95, MW = 1.00, SW = 0.86, UV = 0.45; [14]). The achromatic version of the model was based on the double cones. The output of the model is just noticeable differences (JNDs), whereby JNDs < 1.00 mean that two stimuli are indiscriminable, with higher values indicating that two stimuli should be increasingly distinguishable.

Our statistics tested whether sand fleas are a closer match to the sand from their own beach (lower JNDs) than to sand from other beaches. Analyses were performed in R v. 3.0.2, using generalized mixed linear models specified in lme4 v. 1.1-5 using a Gaussian error structure, with conformity of model assumptions tested using residuals plots, and *p*-values generated by lmerTest v. 2.0-6. A square root transform of JND values best fitted the assumption of normality of error. A full model was first specified where JND was modelled against the interaction between the sand flea beach ID, and whether or not the comparison was with the same beach or a different one (treatment: same or different). The beach ID of the sand flea, and the respective substrate comparison beach ID were also specified in the mixed model as random effects. Models were simplified based on Akaike information criterion weightings [16]. The interaction term was retained in both colour and luminance JND models. Sand flea beach ID was dropped from the models as a random effect, remaining only as a fixed effect.

## 3. Results

**Figure 1.**
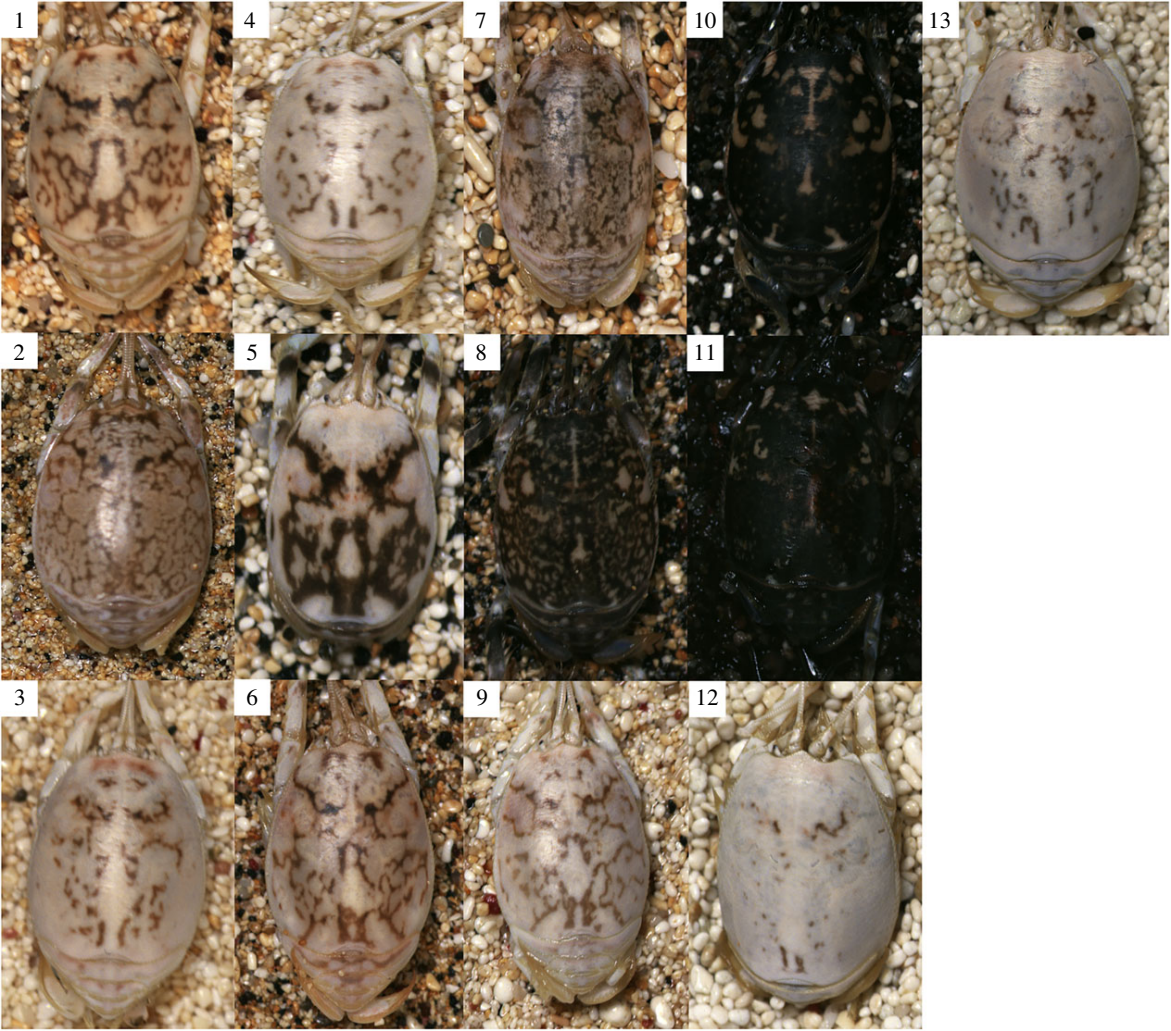
Sand fleas and sand from each of the 13 beaches illustrate the degree of camouflage. Numbers refer to each beach (see the electronic supplementary material).

**Figure 2.**
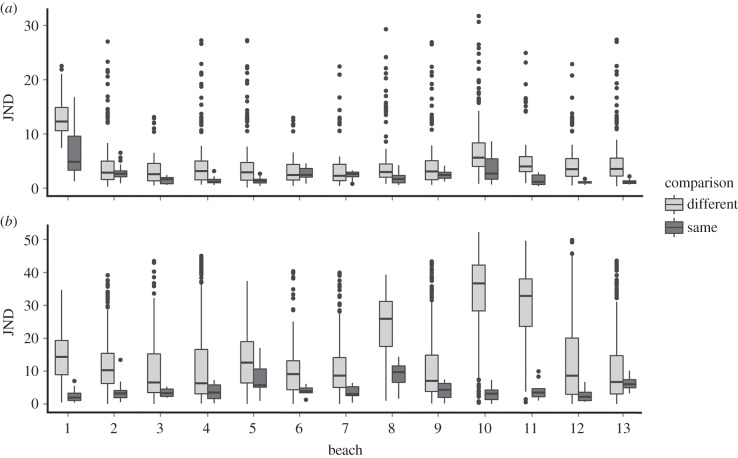
Matching of sand fleas to their own and different beaches for (*a*) colour and (*b*) luminance, with regards to potential avian predators. Camouflage is measured in ‘just noticeable differences' (JNDs), with values less than 1–3 indicating effective camouflage and higher values equating to decreasing matches. Numbers refer to the specific beach.

There were substantial differences between ‘same’ versus ‘different’ beach comparisons. Overall, sand fleas match the colour and luminance of their own substrates (mean ± standard deviation = 2.40 JND ± 2.20 for colour, 4.61 ± 3.30 for luminance) better than those of other beaches (mean 5.32 JND ± 4.91 for colour, 16.75 ± 13.32 for luminance; *F*_1,2613.3_ = 375.2, *p* < 0.001 for colour, and *F*_1,2613.8_ = 322.29, *p* < 0.001 for luminance JNDs; figures 1 and 2). There were also highly significant differences between the level of matching on different beaches, especially for luminance (interaction between same/different and beach ID: *F*_12,2614.1_ = 77.07, *p* < 0.001 for colour, and F_12,2617.7_ = 34.83, *p* < 0.001 for luminance JNDs), with fleas on some beaches better at matching their substrate than fleas on other beaches. Finally, the JND values are very low, indicating effective camouflage. To human eyes, much variation exists in brightness, with very dark individuals on beaches with dark sand, and light yellow individuals on beaches with equivalent appearance. This is reflected in a strong positive relationship between the mean luminance (double cone) values of individuals and their respective beaches (electronic supplementary material, figure S2).

## 4. Discussion

Individual sand fleas from different beaches on Ascension Island differ substantially in colour and brightness, affording them camouflage tuned to the local substrate. Although phenotype–environment associations are thought to be widespread in nature and a common outcome of selection for camouflage, our work provides possibly the first demonstration that this does apparently equate to improved concealment against prospective predators. However, whether this results in reduced predation risk should be tested as such experiments have seldom been undertaken (but see [17]). Although we have based our analyses on avian vision, we cannot discount other predators from targeting individuals, especially fish. Nonetheless, our results should not substantially differ when considering other animal vision; the relationship is clearly apparent through human eyes.

Our study also raises questions regarding the mechanisms that drive substrate-tuned camouflage, both here and in other species. Wenner [7] suggested a number of (not mutually exclusive) mechanisms that could drive his observations in related Pacific mole crabs, including differential survival through selective predation on mismatched morphs, colour change through chromatophore cells, incorporation of pigment into the exoskeleton during moulting and dietary factors. He found evidence that moulting was the primary mechanism by placing individuals on different coloured backgrounds and observing changes in appearance pre- and post-moult (while keeping diet constant). Thus, as seems to be the case with some crabs [4,5], the attainment of camouflage may involve ontogenetic changes. This seems likely given the planktonic-larval stages of these species, which should prevent genetic differentiation at local scales. Most work on colour change in animals has been conducted in species that can change colour relatively rapidly, while comparatively slower changes, and in particular developmental processes, are often neglected (but see [18]). Future work should explore these possibilities and underlying mechanisms, including the role of visual feedback and cellular processes. Ontogenetic changes in coloration with habitat appear widespread in nature, representing a rich opportunity to study both mechanisms and functional aspects of animal appearances.

## Supplementary Material

Supplementary information

## Data accessibility

Data deposited in Dryad: http://dx.doi.org/10.5061/dryad.3ng5c.

## References

[1] Stevens M, Merilaita S 2011 Animal camouflage: from mechanisms to function. Cambridge, UK: Cambridge University Press.

[2] Wallace AR 1867 Mimicry and other protective resemblances among animals. Westminster Rev. (Lond. edn) 1, 1–43.

[3] Nachman MW, Hoekstra HE, D'Agostino SL 2003 The genetic basis of adaptive melanism in pocket mice. PNAS 100, 5268–5273. (10.1073/pnas.0431157100) 12704245 PMC154334

[4] Stevens M, Wood LE, Lown AE 2014 Camouflage and individual variation in shore crabs (*Carcinus maenas*) from different habitats. PLoS ONE 9, e115586 (10.1371/journal.pone.0115586) 25551233 PMC4281232

[5] Todd PA, Briers RA, Ladle RJ, Middleton F 2006 Phenotype-environment matching in the shore crab (*Carcinus maenas*). Mar. Biol. 148, 1357–1367. (10.1007/s00227-005-0159-2)

[6] Rosenblum EB 2006 Convergent evolution and divergent selection: lizards at the White Sands ecotone. Am. Nat. 167, 1–15. (10.1086/498397) 16475095

[7] Wenner AM 1972 Incremental color change in an anomuran decapod *Hippa pacifica* Dana. Pacific Sci 26, 346–353.

[8] Godley BJ, Broderick AC, Glen F, Hays GC 2002 Temperature dependent sex determination of Ascension Island green turtles. Mar. Ecol. Prog. Ser. 226, 115–124. (10.3354/meps226115)

[9] Blokpoel H, Boersma DC, Hughes RA, Tessier GD 1992 Foraging by larids on sand crabs *Emerita analoga* along the coast of southern Peru. Ardea 90, 99–104.

[10] Stevens M, Lown AE, Wood LE 2014 Colour change and camouflage in juvenile shore crabs *Carcinus maenas*. Front. Ecol. Evol. 2, 14 (10.3389/fevo.2014.00014)

[11] Stevens M, Párraga CA, Cuthill IC, Partridge JC, Troscianko TS 2007 Using digital photography to study animal coloration. Biol. J. Linn. Soc. 90, 211–237. (10.1111/j.1095-8312.2007.00725.x)

[12] Troscianko J, Stevens M 2015 Image calibration and analysis toolbox—a free software suite for objectively measuring reflectance, colour and pattern. Methods Ecol. Evol. (10.1111/2041-210X.12439) PMC4791150 27076902

[13] Ödeen A, Håstad O, Alstrom P 2009 Evolution of ultraviolet vision in shorebirds (Charadriiformes). Biol. Lett. 6, 370–374. (10.1098/rsbl.2009.0877) 20015861 PMC2880050

[14] Hart NS 2002 Vision in the peafowl (Aves: *Pavo cristatus*). J. Exp. Biol. 205, 3925–3935. 12432014 10.1242/jeb.205.24.3925

[15] Vorobyev M, Osorio D 1998 Receptor noise as a determinant of colour thresholds. Proc. R. Soc. Lond. B 265, 351–358. (10.1098/rspb.1998.0302) PMC1688899 9523436

[16] Zuur A, Ieno EN, Walker N, Saveliev AA, Smith GM 2009 Mixed effects models and extensions in ecology with R. Berlin, Germany: Springer.

[17] Vignieri SN, Larson JG, Hoekstra HE 2010 The selective advantage of crypsis in mice. Evolution 64, 2153–2158. (10.1111/j.1558-5646.2010.00976.x) 20163447

[18] Detto T, Hemmi JM, Backwell PRY 2008 Colouration and colour changes of the fiddler crab, *Uca capricornis*: a descriptive study. PLoS ONE 3, e1629 (10.1371/journal.pone.0001629) 18286186 PMC2229841

