# Supplementary material for: Phenotype–environment matching in sand fleas
Source: Biol Lett. 2015 Aug;11(8):20150494. doi: 10.1098/rsbl.2015.0494 (PMC4571681; doi:10.1098/rsbl.2015.0494)

**Phenotype-Environment Matching in Sand Fleas**

**Electronic Supplementary Material**

**Sample sizes and collection locations**

**1. Clarkes** (n = 19): approximately straight beach contained by volcanic rock headlands at each end. White biogenic sand mixed with some volcanic dust giving a brownish-orange tinge. Coarse, poorly sorted and sub-angular. (Map ref. 649 194).

**2. Comfortless Cove** (n = 19): small sandy cove enclosed by rock headlands. Yellowish sand comprised of mixed biogenic and volcanic material, medium grained, moderately sorted and sub angular. (Map ref. 658 256).

**3. Deadman’s Beach** (n = 6): substantial beach, approx. 300m long, bounded by two natural rock groynes at either end. White biogenic sand with some shell, coarse, sub-angular, poorly sorted. (Map ref. 643 235).

**4. English Bay** (n = 20): large, sheltered bay, contained by rock headlands. White biogenic sand mixed with some volcanic material, medium to coarse grained, sub-angular and moderately sorted. (Map ref. 680 274)

**5. Hannay’s Beach** (n = 20): medium sized beach backed by steep cliffs and scree slopes. White biogenic sand mixed with black volcanic material (Map. ref. 739 247).

**6. Long Beach A** (n = 5): very large bayhead beach, approx. 970 m long. White biogenic sand mixed with some red volcanic dust giving a slightly orange tinge, some shell material, coarse, poorly sorted, sub-angular. (Map ref. 653 243)

**7. Long Beach B** (n = 11): as above.

**8. North East Bay** (n = 20): large bayhead beach, approx. 330 m long, bounded by two rock headlands. Black-brown volcanic rock sand, fine to medium grained and sub-angular. (Map ref. 724 253)

**9. Pan Am Beach** (n = 20): large, bayhead beach, approx. 500m long backed by vertical cliffs. White biogenic sand, coarse, poorly sorted, sub-angular (Map ref. 649 194).

**10. Pebbly West** (n = 20): small beach bounded by rock headlands at the mouth of a water catchment. Largely stony but with some black, volcanic sand (Map ref. 741 247).

**11. Pebbly East** (n = 10): very small cove at the base of scree slopes. Black volcanic sand (Map ref. 743 245).

**12. Scouts’ Beach** (n = 14): medium size beach, approx. 180m long, bounded by rock headlands. White biogenic sand with a lot of shell. Coarse to very coarse, poorly sorted and sub-angular. (Map ref. 641 226).

**13. Turtle Shell Beach** (n = 20): consists of two tombolos anchored by volcanic rock outcrops. White biogenic sand, coarse, poorly sorted and sub-angular. (Map ref. 645 200).

**Figure ESM 1**: Beach locations on Ascension Island.


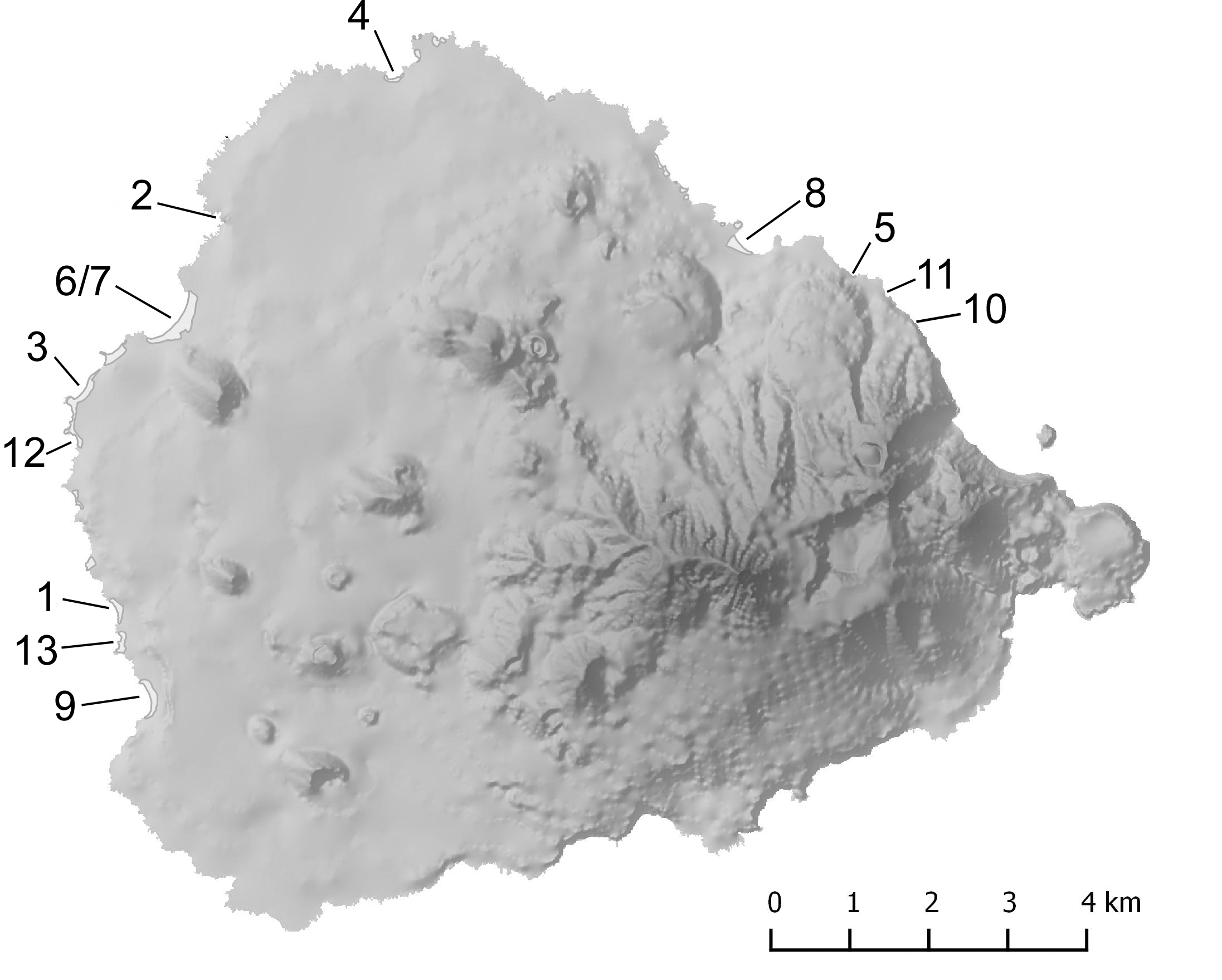


**Figure ESM 2**: Sand fleas show a close match for luminance (in terms of avian double cones) between their own appearance and that of the beach they are from, with a linear regression R^2^ of 0.92. Data show means plus standard deviation bars.


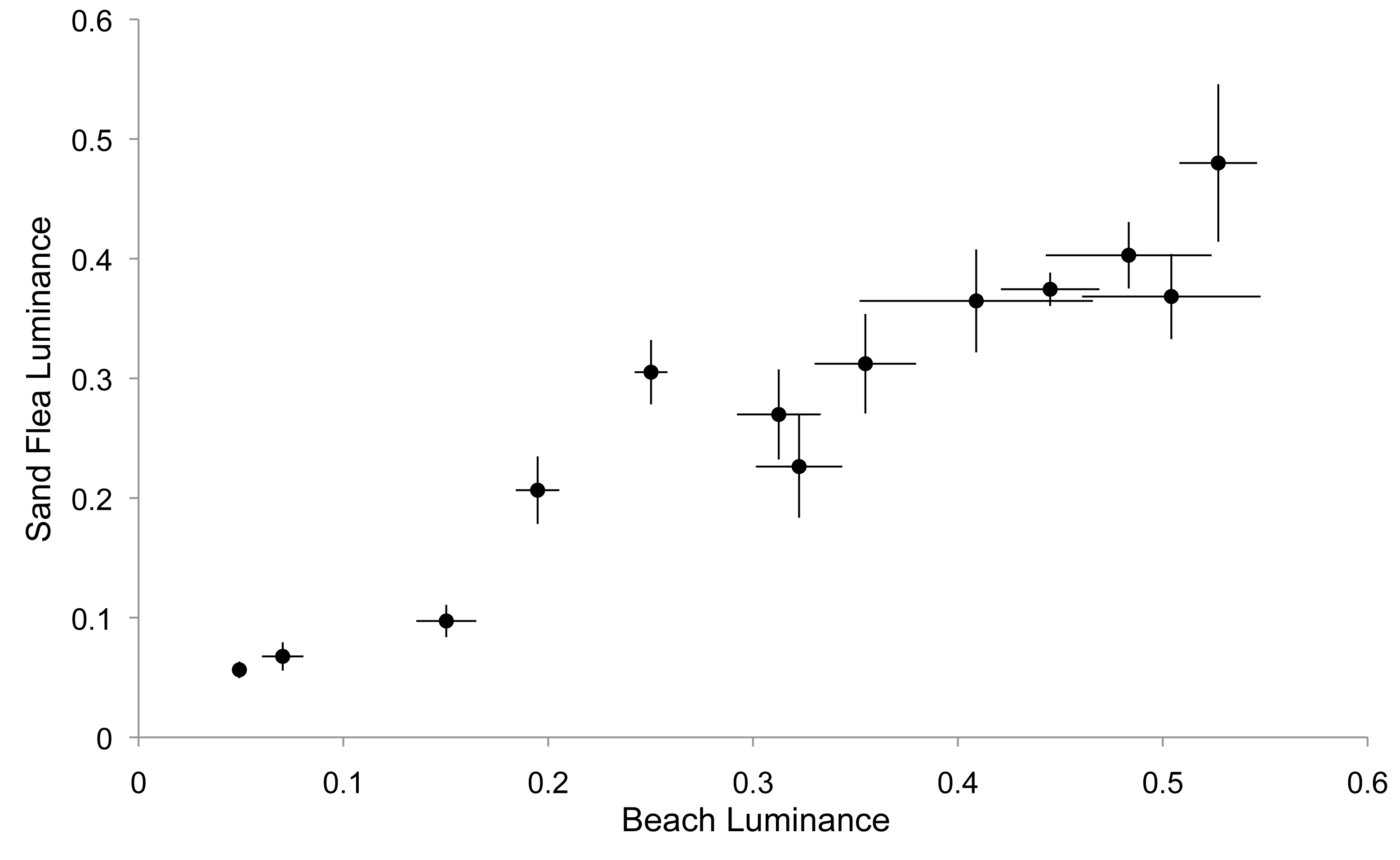

Supplement: Supplementary information [file rsbl20150494supp1.docx]
